# Pannexin1 links lymphatic function to lipid metabolism and atherosclerosis

**DOI:** 10.1038/s41598-017-14130-4

**Published:** 2017-10-20

**Authors:** Filippo Molica, Merlijn J. Meens, Juan Dubrot, Avigail Ehrlich, Christel L. Roth, Sandrine Morel, Graziano Pelli, Laurent Vinet, Vincent Braunersreuther, Osman Ratib, Marc Chanson, Stephanie Hugues, Eliana Scemes, Brenda R. Kwak

**Affiliations:** 1 University of Geneva, Department of Pathology and Immunology, Geneva, CH-1211 Switzerland; 2 Geneva University Hospitals, Department of Radiology and Medical Informatics, Geneva, CH-1211 Switzerland; 3 University of Geneva and Lausanne, School of Pharmaceutical Sciences, Geneva, CH-1211 Switzerland; 4 Geneva University Hospitals and University of Geneva, Department of Pediatrics and of Cell Physiology and Metabolism, Geneva, CH-1211 Switzerland; 5 Albert Einstein College of Medicine, Department of Neuroscience, New York, NY 10461 USA; 6 University of Geneva, Department of Medical Specializations – Cardiology, Geneva, CH-1211 Switzerland

## Abstract

Extracellular ATP is a central signaling molecule in inflammatory responses. Pannexin1 (Panx1) channels release ATP in a controlled manner and have been implicated in various inflammatory pathologies, but their role in atherogenesis remains elusive. Using atherosclerosis-susceptible mouse models with ubiquitous deletion of Panx1 (*Panx1* ^*−/−*^ *Apoe* ^*−/−*^) or with Cre recombinase-mediated deletion of Panx1 in endothelial cells and monocytes (*Tie2-Cre* ^*Tg*^ *Panx1* ^*fl/fl*^ *Apoe* ^*−/−*^; *Panx1* ^*del*^ *Apoe* ^*−/−*^), we identified a novel role for Panx1 in the lymphatic vasculature. Atherosclerotic lesion development in response to high-cholesterol diet was enhanced in *Panx1* ^*del*^ *Apoe* ^*−/−*^ mice, pointing to an atheroprotective role for Panx1 in endothelial and/or monocytic cells. Unexpectedly, atherogenesis was not changed in mice with ubiquitous Panx1 deletion, but *Panx1* ^*−/−*^ *Apoe* ^*−/−*^ mice displayed reduced body weight, serum cholesterol, triglycerides and free fatty acids, suggesting altered lipid metabolism in these Panx1-deficient mice. Mechanistically, *Panx1* ^*−/−*^ *Apoe* ^*−/−*^ mice showed impairment of lymphatic vessel function with decreased drainage of interstitial fluids and reduced dietary fat absorption. Thus, the detrimental effect of Panx1 deletion in endothelial and/or monocytic cells during atherogenesis is counterbalanced by an opposite effect resulting from impaired lymphatic function in ubiquitous Panx1-deficient mice. Collectively, our findings unveil a pivotal role of Panx1 in linking lymphatic function to lipid metabolism and atherosclerotic plaque development.

## Introduction

Atherosclerosis, the leading cause of mortality worldwide, is a chronic immuno-inflammatory disease of large and medium-sized arteries^1^. The disease involves the formation of plaques in the intima of arteries that are characterized by a dysfunctional endothelium, recruitment of leukocytes, lipid accumulation, smooth muscle cell (SMC) migration and proliferation, cell death and fibrosis. The cellular composition of atherosclerotic lesions determines their stability; stable plaques exhibit a thick fibrous cap with many SMCs while the necrotic core size and the number of macrophages are limited^2^. The most severe clinical events, such as myocardial infarction, follow the rupture of an atherosclerotic lesion, which exposes the pro-thrombotic material in the plaque to the blood resulting in thrombus formation and arterial occlusion.

Purinergic signaling in atherosclerosis has recently gained attention. In general, it appears that activation of P2 receptors by adenosine triphosphate (ATP) or other nucleotides promotes atherosclerosis whereas ATP hydrolysis by ecto-nucleotidases to adenosine displays an atheroprotective function^3^. Pannexin1 (Panx1) is important in arterial physiology, mostly through its capacity to form membrane channels that release nucleotides including ATP^4^. As such, Panx1 contributes to the coordination of SMC contraction in resistance arteries and to the endothelium-dependent regulation of arterial tone in conduit arteries^5,6^. Panx1 is also involved in ATP-mediated inflammasome activation, in chemotaxis of neutrophils and in activation of T cells^7^. Finally, Panx1 has been identified as the conduit for ATP from apoptotic cells to release a “find me” signal to recruit phagocytes at the early stages of programmed cell death^8^. Whether Panx1 plays a role in atherosclerotic lesion development and plaque stability remains, however, to be investigated.

## Results and Discussion

### Panx1 deletion in endothelial and monocytic cells promotes atherosclerosis

**Figure 1:**
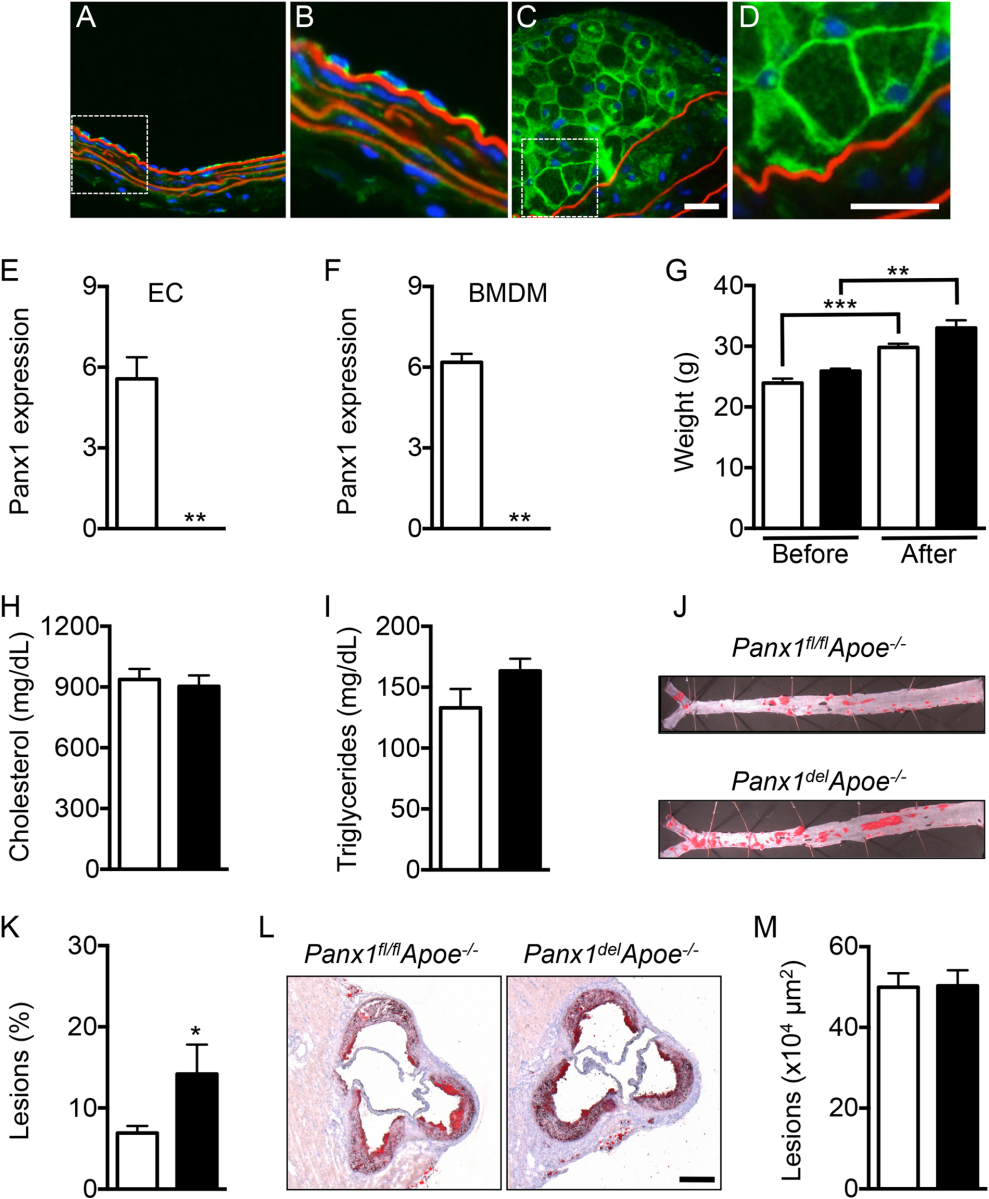
Targeted deletion of Panx1 in endothelial and monocytic cells favors atherosclerotic plaque development. Representative images of Panx1 immunofluorescent staining in ECs of a mouse carotid artery (**A** and **B**) and in macrophage foam cells within a carotid atherosclerotic lesion (**C** and **D**). Nuclei were stained with DAPI (blue) and elastic laminae were visualized with Evans Blue (red). Scale bars represent 50 or 25 μm, respectively. Panx1 expression in ECs (**E**) and BMDMs (**F**) of *Panx1* ^*fl/*fl^ *Apoe* ^*−/−*^ (white bars) and *Panx1* ^*del*^ *Apoe* ^*−/−*^ (black bars) mice was assessed by real-time qPCR (n = 6). (**G**) Weights of *Panx1* ^*fl/fl*^ *Apoe* ^*−/−*^ and *Panx1* ^*del*^ *Apoe* ^*−/−*^ mice before and after 10 weeks of HCD (n = 10). Serum total cholesterol (**H**) and TG (**I**) levels in *Panx1* ^*fl/fl*^ *Apoe* ^*−/−*^ and *Panx1* ^*del*^ *Apoe* ^*−/−*^ mice after 10 weeks of HCD (n = 10). Sudan-IV staining (**J**) and quantification of atherosclerotic lesion extent (**K**) in the thoracic-abdominal aortas and the aortic roots (**L** and **M**) of *Panx1* ^*fl/fl*^ *Apoe* ^*−/−*^ and *Panx1* ^*del*^ *Apoe* ^*−/−*^ mice after 10 weeks of HCD (n = 10). Scale bar represents 200 μm.

We first investigated Panx1 expression in carotid arteries of atherosclerosis-susceptible Apolipoprotein E-deficient (*Apoe* ^*−/−*^) mice fed with high-cholesterol diet (HCD) by immunofluorescent staining. As shown in Fig. 1, non-diseased straight vessel parts showed Panx1 expression exclusively in the endothelium (Fig. 1A and B). Interestingly, an additional strong expression of Panx1 was observed in the membrane of macrophage foam cells in atherosclerotic lesions (Fig. 1C and D). Of note, fluorescent staining was absent from the media of diseased and non-diseased arteries, indicating a lack of Panx1 expression in the SMC layer of large arteries (Fig. 1A–D **)**. Absence of Panx1 in SMCs of large arteries was further confirmed by Western blot using endothelium-denudated aortas from WT and *Panx1* ^*−/−*^ mice (Figure S1A). To investigate a potential contribution of Panx1 in endothelial cells (ECs) and in cells of monocytic origin to atherosclerosis, we generated mice with a conditional deletion of Panx1. Thus, we first interbred *Panx1* ^*fl/fl*^ mice with *Apoe* ^*−/−*^ mice to generate *Panx1* ^*fl/fl*^ *Apoe* ^*−/−*^ mice. Further breeding of these mice with mice harboring the Cre recombinase coding sequence under the control of the 2.1 kb Tie2 promoter^9^ resulted in *Tie2Cre* ^*Tg*^ *Panx1* ^*fl/fl*^ *Apoe* ^*−/−*^ mice (hereafter referred to as *Panx1* ^*del*^ *Apoe* ^*−/−*^). Absence of Panx1 in the endothelium of thoracic-abdominal aortas and in bone marrow-derived macrophages (BMDMs) of *Panx1* ^*del*^ *Apoe* ^*−/−*^ mice was confirmed by real-time polymerase chain reaction (PCR) (Fig. 1E and F). Moreover, absence of Panx1 from ECs of carotid arteries in *Panx1* ^*del*^ *Apoe* ^*−/−*^ mice (Figure S1B) but presence of this protein in the epidermis (Figure S1C) and liver (Figure S1A) further demonstrated the specificity of Panx1 deletion in our *Panx1* ^*del*^ *Apoe* ^*−/−*^ mice. We induced atherosclerosis in control *Panx1* ^*fl/fl*^ *Apoe* ^*−/−*^ and *Panx1* ^*del*^ *Apoe* ^*−/−*^ mice by feeding them from the age of 10 weeks with a HCD for 10 weeks. Mice in both groups gained weight with HCD and no differences were observed between *Panx1* ^*fl/fl*^ *Apoe* ^*−/−*^ and *Panx1* ^*del*^ *Apoe* ^*−/−*^ mice (Fig. 1G). Moreover, total cholesterol and triglyceride (TG) concentration in the serum of mice after HCD did not differ between the two genotypes (Fig. 1H and I **)**. Thoracic-abdominal aortas were longitudinally opened and stained for lipids with Sudan-IV, which is an indicator for the extent of atherosclerosis. We observed increased lipid staining in the thoracic-abdominal aortas of *Panx1* ^*del*^ *Apoe* ^*−/−*^ mice (Fig. 1J and K **)**. In contrast, there were no differences in Sudan-IV staining in aortic sinuses from *Panx1* ^*fl/fl*^ *Apoe* ^*−/−*^ and *Panx1* ^*del*^ *Apoe* ^*−/−*^ mice (Fig. 1L and M **)**. Probably due to a combined action of elevated serum cholesterol and hemodynamic factors, atherosclerotic lesions first appear in aortic sinuses of *Apoe* ^*−/−*^ mice^10^. Hence, differences in atherogenesis due to genes important in the early phases of the disease might be plateaued out in the more advanced plaques in the aortic sinus after 10 weeks of HCD. To test this hypothesis, *Panx1* ^*fl/fl*^ *Apoe* ^*−/−*^ and *Panx1* ^*del*^ *Apoe* ^*−/−*^ mice were placed on a HCD for 5 weeks. Lipid staining in thoracic-abdominal aortas confirmed increased atherosclerotic plaque development in *Panx1* ^*del*^ *Apoe* ^*−/−*^ mice after this shorter period of HCD (Figure S2). Altogether, these results point to a protective role for Panx1 expression in the endothelium and/or monocytes/macrophages in atherosclerosis.

### Ubiquitous Panx1 deletion affects serum lipid levels

Panx1 is a widely expressed protein and its expression is up-regulated under acute inflammatory conditions of variable origin^4^. Moreover, general blockade of Panx1 channels with the Food and Drug Administration (FDA)-approved drugs probenecid or mefloquine has been shown effective in mice in the context of acute *P. aeruginosa* pneumonia^11^, in acute cerebral ischemia-reperfusion injury^12^ and in a rodent model of multiple sclerosis known as experimental autoimmune encephalomyelitis^13^. To investigate whether ubiquitous deletion of Panx1 affects atherosclerosis in mice, we placed 10 weeks-old *Panx1* ^*−/−*^ *Apoe* ^*−/−*^ and *Apoe* ^*−/−*^ mice on a HCD for 10 weeks and analyzed atherosclerotic lesion development. Surprisingly, lesion size was not affected by genotype both at the level of the aortic sinus and the thoracic-abdominal aorta (Figure S3A–D). It has previously been reported that *Panx1* ^*−/−*^ mice generated with the knockout (KO)-first strategy may show some remaining Panx1 mRNA expression^14^, although this did not alter the protection that these KO-first *Panx1* ^*−/−*^ mice displayed against seizures as compared to a mouse *Panx1* ^*−/−*^ line of another origin^15^. Similarly, we found about 30% remaining Panx1 mRNA or protein in samples extracted from *Panx1* ^*−/−*^ *Apoe* ^*−/−*^ bladders or kidneys, organs known to express relatively high levels of Panx1 (Figure S4A–C **)**. However, Panx1 protein was absent in atherosclerotic lesions of *Panx1* ^*−/−*^ *Apoe* ^*−/−*^ mice whereas a strong Panx1 signal was detected in the endothelium and macrophages in atherosclerotic lesions of control *Apoe* ^*−/−*^ mice (Figure S4D–G). Thus, the lack of effect on atherosclerosis in *Panx1* ^*−/−*^ *Apoe* ^*−/−*^ mice is likely not caused by remaining Panx1 protein expression.

**Figure 2:**
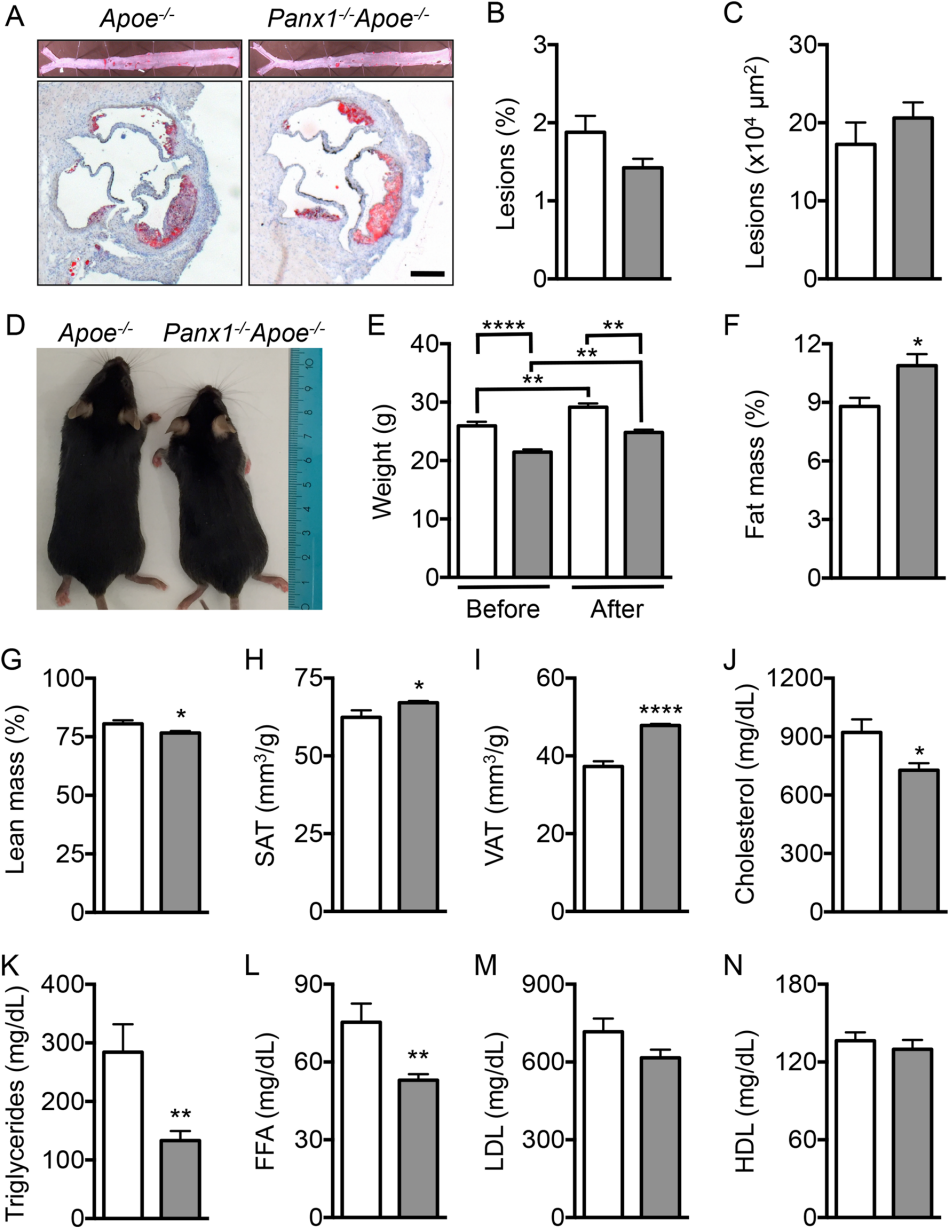
Ubiquitous Panx1 deletion does not affect atherogenesis. Sudan-IV staining (**A**) and quantification of atherosclerotic lesion extent in the thoracic-abdominal aortas (**B**) and in the aortic roots (**C**) of *Apoe* ^*−/−*^ (white bars) and *Panx1* ^*−/−*^ *Apoe* ^*−/−*^ (grey bars) mice after 5 weeks of HCD (n = 10). Scale bar represents 200 μm. (**D**) Representative image illustrating the difference in size between *Apoe* ^*−/−*^ and *Panx1* ^*−/−*^ *Apoe* ^*−/−*^ mice at the age of 10 weeks (n = 10). (**E**) Weights of *Apoe* ^*−/−*^ and *Panx1* ^*−/−*^ *Apoe* ^*−/−*^ mice before and after 5 weeks of HCD (n = 10). Fat mass (**F**) and lean mass (**G**) of *Apoe* ^*−/−*^ and *Panx1* ^*−/−*^ *Apoe* ^*−/−*^ mice were measured by MRI (n = 5). Subcutaneous adipose tissue (SAT; **H**) and visceral adipose tissue (VAT; **I**) in *Apoe* ^*−/−*^ and *Panx1* ^*−/−*^ *Apoe* ^*−/−*^ mice was determined by micro X-ray computed tomography (n = 10). Serum total cholesterol (**J**), TG (**K**), FFA (**L**), LDL (**M**) and HDL (**N**) levels in *Apoe* ^*−/−*^ and *Panx1* ^*−/−*^ *Apoe* ^*−/−*^ mice were measured after 5 weeks of HCD (n = 10).

Reasoning that endothelial and monocytic Panx1 expression appeared protective early in atherosclerotic plaque development, we then examined atherosclerotic lesion development in *Panx1* ^*−/−*^ *Apoe* ^*−/−*^ and *Apoe* ^*−/−*^ mice after only 5 weeks of HCD. As expected, only few and small plaques were observed in the thoracic-abdominal aortas and aortic sinuses at this early time point (Fig. 2A), however, we could still not observe any difference in atherogenesis between both genotypes (Fig. 2B and C). Although the weight gain after 5 weeks HCD was similar in *Panx1* ^*−/−*^ *Apoe* ^*−/−*^ and *Apoe* ^*−/−*^ mice, *Panx1* ^*−/−*^ *Apoe* ^*−/−*^ mice were significantly smaller than *Apoe* ^*−/−*^ controls both before and after HCD (Fig. 2D and E), suggesting metabolic differences between control mice and mice lacking Panx1. In keeping with this idea, magnetic resonance imaging revealed that *Panx1* ^*−/−*^ *Apoe* ^*−/−*^ mice had an increased fat mass and a decreased lean mass compared to their *Apoe* ^*−/−*^ counterparts (Fig. 2F and G). Micro X-ray computed tomography on the same mice revealed that both the subcutaneous and the visceral adipose tissue were significantly increased in *Panx1* ^*−/−*^ *Apoe* ^*−/−*^ mice (Fig. 2H and I and Movie S1 (*Apoe* ^*−/−*^) and S2 (*Panx1* ^*−/−*^ *Apoe* ^*−/−*^)). Measurements of total serum cholesterol, TG and free fatty acids (FFA) revealed that the levels of these parameters were much lower in *Panx1* ^*−/−*^ *Apoe* ^*−/−*^ mice (Fig. 2J–L) whereas LDL and HDL levels were not different from *Apoe* ^*−/−*^ controls (Fig. 2M and N). It is well known that lowering serum cholesterol and TG levels protects against atherosclerosis^16^, thus the lack of effect of ubiquitous deletion of Panx1 on the extent of atherosclerosis is most likely explained by simultaneous opposite effects of Panx1 on lipid metabolism and inflammation. As the extent of atherosclerosis is however not necessarily linked to the vulnerability of lesions for rupture^17^, it remains crucial to study the effects of Panx1 deletion on plaque stability.

### Panx1 deletion affects atherosclerotic lesion phenotype

**Figure 3:**
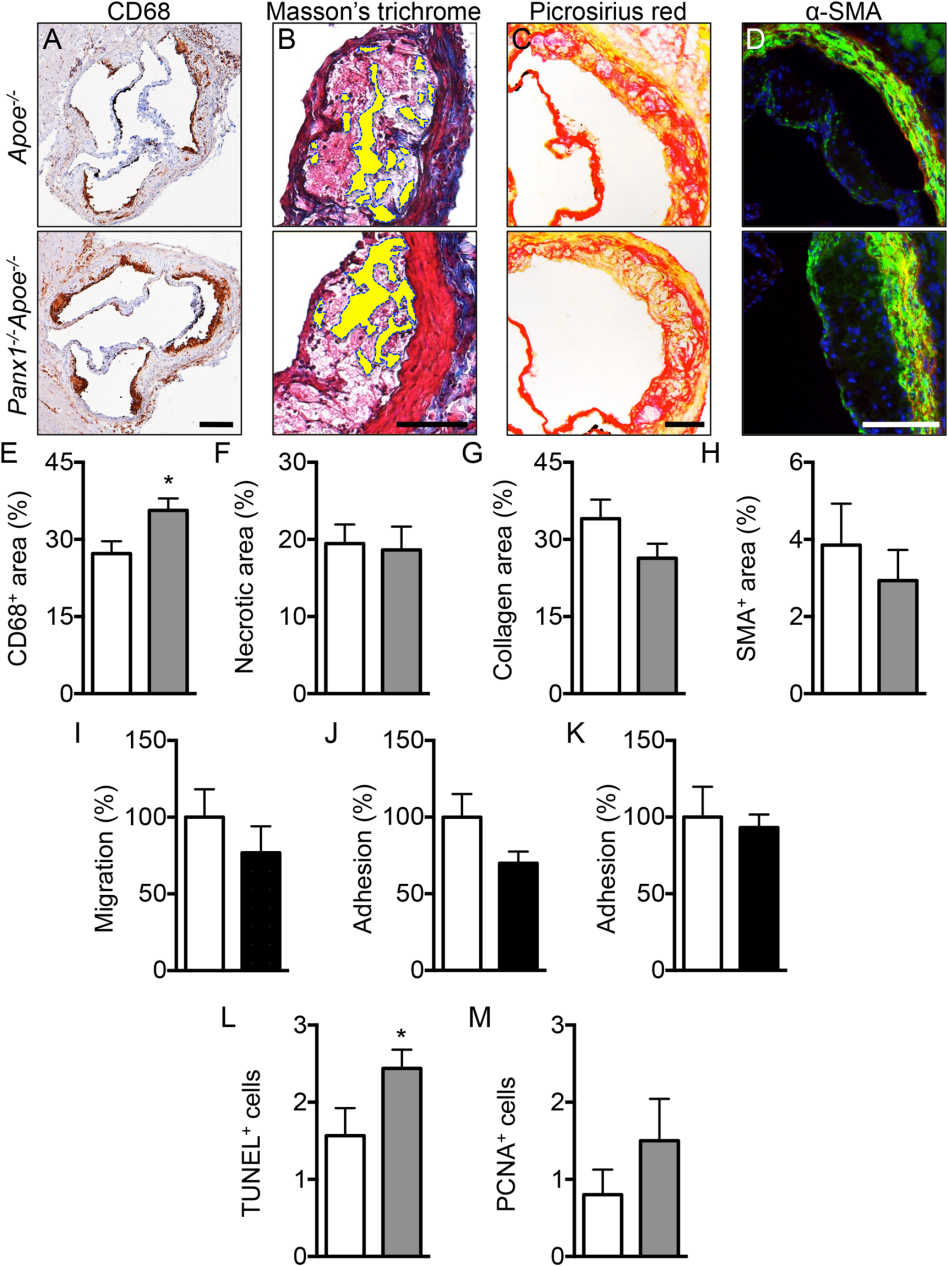
Reduced atherosclerotic plaque stability in mice lacking Panx1. Representative images and quantification of (immuno-)stainings for CD68 (**A**,**E;** brown signal), necrotic core (**B**,**F;** yellow-marked acellular areas), collagen (**C**,**G;** red signal) and α-SMA (**D**,**H;** green signal) performed on aortic roots of *Apoe* ^*−/−*^ (white bars) and *Panx1* ^*−/−*^ *Apoe* ^*−/−*^ (grey bars) mice after 5 weeks of HCD (n = 10). MCP-1-induced chemotaxis (**I**) and adhesion onto collagen-coated (**J**) or fibronectin-coated (**K**) surfaces of *Panx1* ^*fl/fl*^ *Apoe* ^*−/−*^ (white bars) and *Panx1* ^*del*^ *Apoe* ^*−/−*^ (black bars) BMDMs (n = 6). Number of apoptotic cells (**L**) using TUNEL staining or proliferating cells (**M**) using PCNA staining performed on aortic roots of *Apoe* ^*−/−*^ (white bars) and *Panx1* ^*−/−*^ *Apoe* ^*−/−*^ (grey bars) mice after 5 weeks of HCD (n = 10). Scale bars represent 200 μm for **A**, or 100 μm for **B**,**C**, and **D**.

Rupture of atherosclerotic lesions may result from a loss of their mechanical stability, *i.e*. reduced tensile strength of the collagen cap covering the lesion and lipid core accumulation^18^. Indeed, histopathological analysis of plaques that have provoked fatal myocardial infarction show a large lipid core and reduced thickness of the fibrous cap, an accumulation of activated macrophages that produce enzymes digesting fibrillar collagen such as matrix metalloproteinases and a depletion of SMCs that synthesize arterial extracellular matrix^18^. Thus, we assessed whether Panx1 affects the phenotype of atherosclerotic lesions by exploring the plaque components in *Panx1* ^*−/−*^ *Apoe* ^*−/−*^ and *Apoe* ^*−/−*^ mice after 5 weeks of HCD. As shown in Fig. 3A and E, immunostainings for macrophages revealed that the CD68^+^ area was significantly larger in atherosclerotic lesions of *Panx1* ^*−/−*^ *Apoe* ^*−/−*^ mice compared to controls, but no differences were found in lipid/necrotic core size (Fig. 3B and F), collagen content (Fig. 3C and G) and SMC content (Fig. 3D and H) in the lesions of the two groups of mice. To investigate the effects of Panx1 deletion on plaque phenotype in more detail, we used shear stress-modifying casts known to induce atherosclerotic plaques with a stable (induced by oscillatory shear stress (OSS)) and vulnerable (induced by low laminar shear stress (LLSS)) phenotype in common carotid arteries of *Apoe* ^*−/−*^ mice^19,20^. As expected, the CD68^+^ area was increased in plaques induced by LLSS as compared to lesions induced by OSS in *Apoe* ^*−/−*^ mice, whereas an inverted pattern was observed for SMC content of these plaques (Figure S5A–D). Interestingly, such differences in plaque vulnerability markers were not found between LLSS- and OSS-induced atherosclerotic lesions in *Panx1* ^*−/−*^ *Apoe* ^*−/−*^ mice (Figure S5A–D), suggesting that Panx1 deletion abrogated the development of a stable plaque phenotype under OSS. This effect seemed mostly due to reduced presence of SMCs in these lesions (Figure S5C,D), although the CD68^+^ area tended to be augmented as well (Figure S5A,B). Activated macrophages in atherosclerotic lesions express high levels of CD68 although this marker is also present in dendritic cells (DCs)^21^ and SMC-derived macrophages^22^. Therefore, we performed additional immunostainings using F4/80, a more specific marker for mature macrophages^22^, and observed a larger F4/80^+^ area in OSS-induced plaques of *Panx1* ^*−/−*^ *Apoe* ^*−/−*^ mice compared with *Apoe* ^*−/−*^ mice (Figure S5E). Finally, LLSS-induced plaques in *Panx1* ^*−/−*^ *Apoe* ^*−/−*^ mice displayed a larger necrotic core area than the lesions in *Apoe* ^*−/−*^ mice (Figure S5F) and no significant differences were found in the plaque collagen content between both genotypes (Figure S5G). Altogether, these results suggest that Panx1 contributes to the development of a more stable plaque phenotype through a reduction in the size of the necrotic core and in the macrophage content as well as an increase in the SMC content.

To evaluate whether Panx1 affects macrophage recruitment, we first performed chemotaxis experiments using BMDMs from *Panx1* ^*del*^ *Apoe* ^*−/−*^ and *Panx1* ^*fl/fl*^ *Apoe* ^*−/−*^ control mice. In agreement with earlier studies on *Panx1* ^*−/−*^ macrophages^23,24^, deletion of Panx1 from BMDMs (Fig. 1F) did not alter the chemotactic response to monocyte chemoattractant protein (MCP)-1 in these cells (Fig. 3I). Then, we tested the ability of these BMDMs to adhere to collagen-coated or fibronectin-coated surfaces. Adhesion to collagen or fibronectin was similar in BMDMs from both genotypes (Fig. 3J and K), thus it is unlikely that increased macrophage recruitment to atherosclerotic lesions would account for the increased F4/80^+^ content observed in plaques of Panx1-deficient mice (Figure S5E). In addition, these experiments point to a role for endothelial Panx1 rather than Panx1 in monocytic cells in early atherosclerotic plaque development (Fig. 1K). Alternatively, Panx1 may play a role in proliferation, apoptosis or clearance of macrophages from atherosclerotic lesions. Indeed, apoptotic cells release a “find me” signal to recruit phagocytes at the early stages of programmed cell death and Panx1 was identified as the conduit for ATP release from such apoptotic cells^8^. We therefore counted the number of apoptotic cells and proliferating cells in atherosclerotic plaques of *Panx1* ^*−/−*^ *Apoe* ^*−/−*^ and *Apoe* ^*−/−*^ mice using terminal deoxynucleotidyl transferase dUTP nick end labeling (TUNEL) and proliferating cell nuclear antigen (PCNA) assays, respectively. As shown in Fig. 3L and M, we detected only few apoptotic and proliferating cells in atherosclerotic lesions in the aortic roots of *Panx1* ^*−/−*^ *Apoe* ^*−/−*^ and *Apoe* ^*−/−*^ mice. Moreover, no remarkable changes in the number of apoptotic and proliferating cells were detected in atherosclerotic lesions between both genotypes (Fig. 3L and M). Taken together, these results indicate that increased F4/80^+^ content, and likely the increased CD68^+^ content, in atherosclerotic plaques of *Panx1* ^*−/−*^ *Apoe* ^*−/−*^ mice is not due to a raise in chemotactic, adhesive, apoptotic or proliferative capacities of macrophages. This leads to the hypothesis that Panx1 may negatively regulate the exit of CD68^+^-F4/80^+^ cells from atherosclerotic lesions resulting in a more vulnerable plaque phenotype. The importance of the lymphatic vessels in removing immune cells and cholesterol from atherosclerotic plaques is increasingly recognized^25^.

### Ubiquitous Panx1 deletion alters lymphatic function

**Figure 4:**
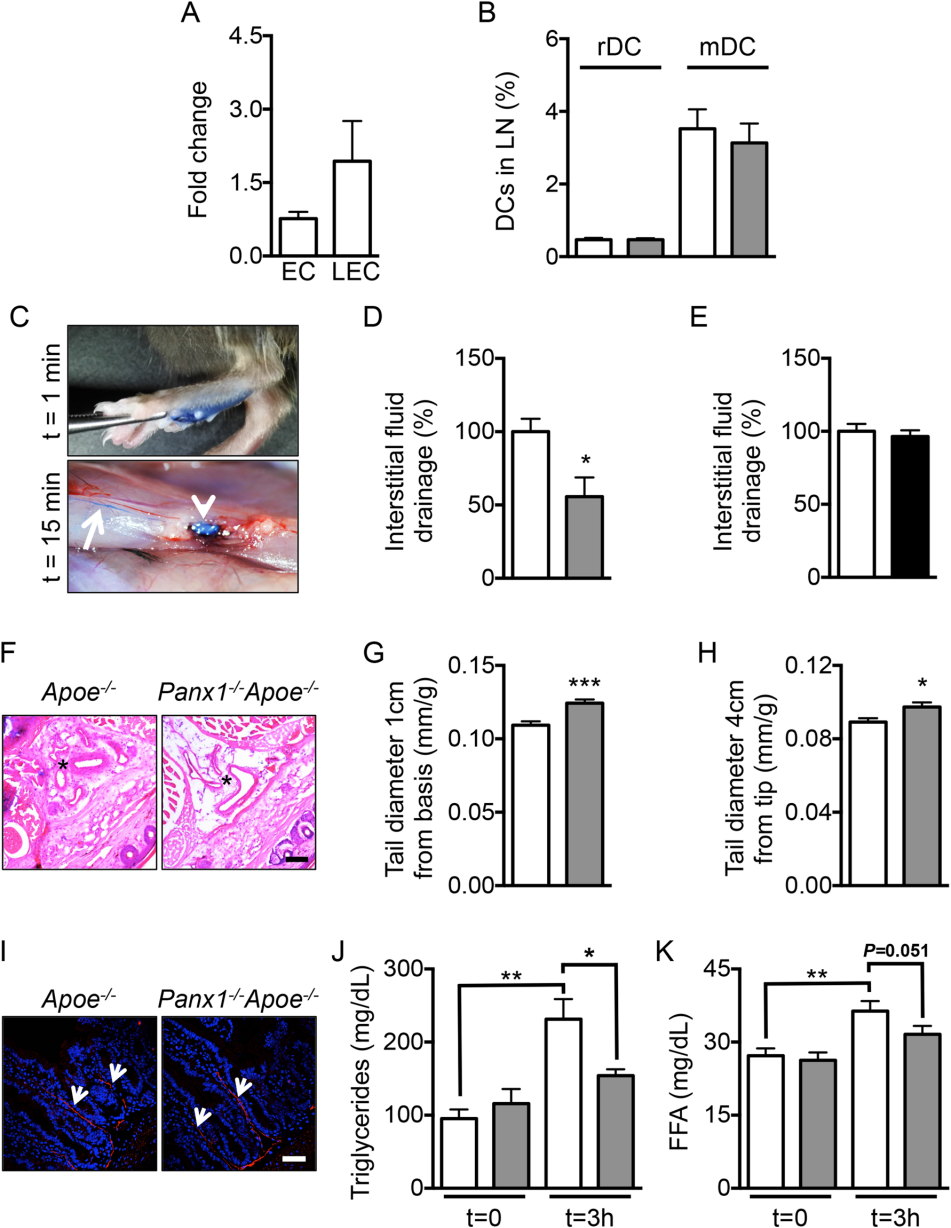
Panx1 deficiency reduces lymphatic function. (**A**) Panx1 expression in aortic ECs and LECs from WT mice was assessed by real-time qPCR (n = 3–4). (**B**) Percentage of resident and migratory DCs in CH draining lymph nodes of control (white bars) and *Panx1* ^*−/−*^ mice (grey bars) (n = 5). (**C**) Representative images of lymphatic drainage 1 and 15 min after injection of 5 μl of Evans Blue. Arrow points to lymphatic vessel and arrowhead to lymph node. Lymphatic function was measured by quantification of Evans Blue in the sera of *Apoe* ^*−/−*^ (white bar) and *Panx1* ^*−/−*^ *Apoe* ^*−/−*^ (grey bar) mice (**D**; n = 4), and of *Panx1* ^*fl/fl*^ *Apoe* ^*−/−*^ (white bar) and *Panx1* ^*del*^ *Apoe* ^*−/−*^ (black bar) mice (**E**; n = 6). (**F**) Representative images of Hematoxylin/Eosin stained cryosections of tails (1 cm from top) from *Apoe* ^*−/−*^ and *Panx1* ^*−/−*^ *Apoe* ^*−/−*^ mice. Asterisks denote regions rich in microvasculature. Scale bar represents 100 μm. Tail diameter quantification in *Apoe* ^*−/−*^ (white bars) and *Panx1* ^*−/−*^ *Apoe* ^*−/−*^ (grey bars) mice was measured at (**G**) 1 cm from the basis and at (**H**) 4 cm from the tip of the tail (n = 16–19). (**I**) LYVE-1 immunostaining (red; arrows) in intestinal villi of *Apoe* ^*−/−*^ and *Panx1* ^*−/−*^ *Apoe* ^*−/−*^ mice. Nuclei were stained with DAPI (blue). Scale bar represents 50 μm. TG (**J**) and FFA (**K**) concentration measured before and 3 hours after olive oil gavage of *Apoe* ^*−/−*^ (white bars) and *Panx1* ^*−/−*^ *Apoe* ^*−/−*^ (grey bars) mice (n = 6).

The lymphatic system forms a unidirectional transport pathway from the interstitial space to systemic veins regulating tissue fluid homeostasis, absorption of dietary fat and trafficking of antigen-presenting cells to draining lymph nodes (LNs)^26^. Macrophages express high levels of CD68 although this marker is also present in dendritic cells (DCs). DCs and to a lesser extent macrophages are triggered by inflammatory stimuli to exit from tissues to draining LNs where they present peptides on major histocompatibility complex molecules to initiate T cell responses^27^. Similar to ECs in blood vessels, lymphatic ECs (LECs) express Panx1 (Fig. 4A). Panx1 was also detected in a lymphatic endothelial cell line (LyEnd.5^28^; data not shown). Lymphatic ECs are important in the trafficking of DCs to draining LNs by promoting DC entry into lymphatic vessels and by regulating their intra-lymphatic motility. To investigate whether Panx1 affects trafficking of DCs, we used a model of contact hypersensitivity (CH) to enhance migration of skin DCs to draining LNs^29^. Twenty-four hours after exposure of the skin in the right flank to acetone/dibutyl phthalate, cells were isolated from draining and non-draining LNs. T cells and B cells represented each about 40% (data not shown) and residential DCs less than 1% of the total cell population in draining and non-draining LNs of both *Panx1* ^*−/−*^ and control wild-type mice (Fig. 4B). The percentage of migratory DCs arriving in CH draining LNs was also comparable in *Panx1* ^*−/−*^ mice and control mice (Fig. 4B), suggesting that Panx1 is not required for regulatory DC – LEC interactions. In contrast, Panx1 channels are known to regulate leukocyte emigration through the venous endothelium during acute inflammation, a process that involves activation of endothelial type-1 tumor necrosis factor (TNF) receptors, recruitment of Src family kinases (SFK) and SFK-dependent phosphorylation of Panx1, ATP release and subsequent activation of P2Y receptors^30^.

To investigate whether Panx1 affects lymphatic flow, we compared drainage of interstitial fluids following injection of Evans Blue in the left footpad of *Panx1* ^*−/−*^ *Apoe* ^*−/−*^ and *Apoe* ^*−/−*^ mice^31^. The dye progressively spreads throughout the lymphatic system to successive draining LNs (Fig. 4C). We collected sera 15 min after injection and the amount of dye was quantified. As illustrated in Fig. 4D, Evans Blue transport to the systemic circulation of *Panx1* ^*−/−*^ *Apoe* ^*−/−*^ mice was considerably smaller than in control *Apoe* ^*−/−*^ mice, suggesting that lymphatic flow is impaired in mice with ubiquitous deletion of Panx1. Moreover, lymphatic vessel density was not different in the ears of *Panx1* ^*−/−*^ *Apoe* ^*−/−*^ and *Apoe* ^*−/−*^ mice (Figure S6). Although mammalian lymphatic vasculature was originally thought to exclusively form by sprouting from embryonic veins, recent studies using Tie2 lineage tracking demonstrate a significant contribution of non-venous derived cells to the lymphatic vasculature^32^. In accordance, we did not find any difference in drainage of Evans Blue through lymphatics in *Panx1* ^*del*^ *Apoe* ^*−/−*^ as compared to *Panx1* ^*fl/fl*^ *Apoe* ^*−/−*^ control mice (Fig. 4E). As compared to wild-type mice, *Apoe* ^*−/−*^ mice exhibit tail swelling resulting from impaired interstitial fluids drainage^33^. To test whether Panx1 deficiency may exacerbate this effect, we measured tail diameters in *Apoe* ^*−/−*^ and *Panx1* ^*−/−*^ *Apoe* ^*−/−*^ mice. Hematoxylin/Eosin stainings on cryosections of *Panx1* ^*−/−*^ *Apoe* ^*−/−*^ tails suggested increased interstitial fluid content, in particular around the microcirculation, as compared to *Apoe* ^*−/−*^ tails (Fig. 4F). Indeed, diameters measured at 1 cm from the basis (Figs. 4G) or 4 cm from the tip of the tail (Fig. 4H) were increased in *Panx1* ^*−/−*^ *Apoe* ^*−/−*^ mice, confirming impaired drainage of interstitial fluids in absence of Panx1. Together, these results suggest that Panx1 expression in lymphatic endothelium contributes to the drainage of interstitial fluid.

In view of these results, the reduced total cholesterol, TG and FFA serum values in *Panx1* ^*−/−*^ *Apoe* ^*−/−*^ mice after HCD (Fig. 2J–L) might be caused by defective uptake or degradation of TG-rich remnants, such as chylomicrons. Lymphatic capillaries in the center of the villi in the small intestine, called lacteals, play a primary role in the absorption of dietary fat and fat-soluble vitamins^26^. As shown in Fig. 4I, lacteals were readily identified by LYVE-1 staining in the jejunum of both *Panx1* ^*−/−*^ *Apoe* ^*−/−*^ and *Apoe* ^*−/−*^ mice. To study chylomicron absorption, we gavaged *Panx1* ^*−/−*^ *Apoe* ^*−/−*^ and *Apoe* ^*−/−*^ mice with olive oil and measured accumulation of plasma TG and FFA 3 hours later. *Panx1* ^*−/−*^ *Apoe* ^*−/−*^ mice had a blunted increase in plasma TG and a trend towards reduced FFA levels compared with control animals (Fig. 4J and K). As expected, total cholesterol levels remained unchanged in both genotypes (data not shown). Together, these results indicate that Panx1 in lacteals contributes to the uptake of dietary fat from the gut. Whether additional factors, such as feeding behavior, energy expenditure or lipoprotein breakdown, further contribute to the observed weight and TG differences between *Panx1* ^*−/−*^ *Apoe* ^*−/−*^ and *Apoe* ^*−/−*^ mice remains to be investigated. Of note, selective ablation of Panx1 from adipocytes only modestly enhances parameters of insulin resistance, including glucose and insulin tolerance, after 12 weeks of diabetogenic diet without affecting weight, adiposity or FFA levels of the mice^34^.

The role of the lymphatic system in the regulation of immune responses has been extensively studied, but research towards the role of this system in atherosclerotic disease is just at its infancy. Vuorio and colleagues reported increased plasma cholesterol levels and enhanced atherogenesis in independent mouse models with severe lymphatic insufficiency, which suggests a role for lymphatic vessels in the maintenance of serum lipoprotein balance and vascular homeostasis^35^. However, another recent study points to the lymphatic system as a critical player for removing cholesterol from atherosclerotic lesions by reverse cholesterol transport^36^. The reason for this discrepancy is not clear and remains to be investigated, one could for example hypothesize that the lymphatic system plays different roles in early and advanced stages of atherosclerosis. Interestingly, both in mice and human, lymphatic vessels are mostly present in the adventitial layer of arterial walls, and arteries with a dense lymphatic network seem naturally protected against atherosclerosis^25^, thus further supporting lymphatic transport function may improve cholesterol clearance in therapies aimed at reversing atherosclerosis. In the present study, we have identified Panx1 as a novel regulator of lymphatic function, thereby determining lipid metabolism as well as atherosclerotic plaque development and stability. Our knowledge on compounds precisely regulating the open probability of Panx1 channels, including already FDA-approved drugs, is rapidly increasing and holds promises for new therapeutic strategies in the prevention or treatment of atherosclerosis, a disease that is still the number one cause of mortality in industrialized countries.

## Methods

### Animals

All animal studies were performed after approval by the Swiss Federal Veterinary Office and were in accordance with the established Swiss guidelines and regulations. For atherosclerosis studies, we used 10 weeks-old male *Panx1* ^*−/−*^ *Apoe* ^*−/−*^ *, Apoe* ^*−/−*^, *Tie2Cre* ^*Tg*^ *Panx1* ^*fl/fl*^ *Apoe* ^*−/−*^ (*Panx1* ^*del*^ *Apoe* ^*−/−*^) and *Panx1* ^*fl/fl*^ *Apoe* ^*−/−*^ mice, on a C57BL/6 background, fed with HCD (1.25% cholesterol, 0% cholate; Research Diets Inc.) for 5 or 10 weeks. Mice were euthanized after general anesthesia induced by an intraperitoneal injection of ketarom (10 mg/kg xylazine mixed with 100 mg/kg ketamine). Blood was taken and mice were perfused with 0.9% NaCl. Aortic roots and pieces of flank skin were embedded in OCT compound (Tissue-Tek; Sakura) and snap-frozen. Carotid arteries and thoracic-abdominal aortas were fixed in 4% paraformaldehyde (PFA) for 2 hours. Carotids were then immerged overnight (ON) in 30% sucrose before being snap-frozen in OCT compound. Thoracic-abdominal aortas were rinsed with phosphate-buffered saline (PBS) and incubated overnight in Sudan-IV solution for lipid deposition analysis as described previously^37^. The vessels were opened longitudinally and the extent of atherosclerosis was determined by dividing Sudan-IV-positive area by the total surface of the thoracic-abdominal aorta. Atherosclerotic plaque formation was also assessed in aortic roots. For each root an average lesion area was quantified after Sudan-IV staining of 6 serial cross-sections (5 μm thickness, inter-distance 50 μm). Images were captured with Zeiss Axiophot microscope and quantification was performed by computer image analysis using Image J.

### *In vivo* alteration of shear stress

A conical shear stress-modifying cast was used to induce defined changes in shear stress, as previously described^19,20^. In brief, 10 weeks-old male *Panx1* ^*−/−*^ *Apoe* ^*−/−*^ *and Apoe* ^*−/−*^ mice were anesthetized with 5% isoflurane inhalation for induction, followed by 2% isoflurane for maintenance of anesthesia. The anterior cervical triangle was accessed by a sagittal anterior neck incision. Both halves of the vascular cast were placed around the right common carotid artery and fixed with a suture. Post-operative analgesia was performed with intraperitoneal injection of Buprenorphinum (0.05 mg/kg) for 3 days. Mice were fed a HCD and killed 10 weeks after cast placement following general anesthesia with ketamine 100 mg/kg and xylazine 10 mg/kg i.p. After perfusion with 0.9% NaCl, casted vessels were excised and the cast was removed. Samples were embedded in OCT compound and snap frozen. Five μm-thick serial cryosections were obtained from the 3 flow regions determined by the conical, progressively constrictive shape of the cast, *i.e*. upstream – LLSS, inside – high laminar shear stress (HLSS), downstream – OSS.

### Histological analysis

Immunofluorescence: Cryosections (5 μm) of atherosclerotic lesions in carotids of 10 weeks-old male mice fed with a HCD were fixed 15 min in 4% PFA, permeabilized 15 min with 0.3% Triton X-100 and blocked 30 min in PBS containing 2% bovine serum albumin (BSA). Chicken anti-mouse Panx1_414–425_ antibodies^38^ (1:500) were then applied ON on the sections before detection with goat anti-chicken DyLight488 antibody (1:500; Jackson Laboratories) for 2 hours at RT and subsequent Evans Blue counterstaining. A similar protocol was applied to cryosections (5 μm) of flank skin. Moreover, 4% PFA-fixed jejunum cryosections (5 μm) were permeabilized 60 min with 0.2% Triton X-100 in PBS and blocked 30 min in PBS-BSA (2%). LYVE-1 antibody (1:50; Abcam) was incubated 1 hour. Secondary goat anti-rabbit Alexa 568 antibody (1:2000; Jackson Laboratories) was then incubated 1 hour at RT. All sections were counterstained with 4′,6-diamidine-2′-phenylindole dihydrochloride (DAPI), mounted with Vectashield medium (Vector Laboratories) and analysed using a Zeiss Axioskop 2 microscope or a Zeiss LSM700 confocal microscope.

Immunostainings on cryosections of aortic roots or casted carotids. For macrophages: ON incubation at 4 °C of 100% acetone-fixed sections with CD68 antibody (1:100; Bio-Rad) followed by a 1 hour incubation at RT with corresponding horse radish peroxidase-conjugated antibody (1:500; Jackson Laboratories). Alternatively, macrophage staining was performed after a 2 hour incubation at RT of 4% PFA-fixed sections with F4/80 antibody (1/100; Caltag) followed by 1 hour incubation at RT with an Alexa 488 antibody (1:2000; Jackson Laboratories). For SMCs: α-SMA antibody^39^ (1:50) was incubated for 1 hour at RT on 4% PFA-fixed sections and detected by incubating with a goat anti-mouse IgG2a Alexa 488 antibody (1:100; Jackson Laboratories) for 30 min at RT. For proliferation: PCNA antibody (1:20; ThermoScientific) was incubated ON at 4 °C on sections fixed successively at RT with 4% PFA (15 min) and methanol (5 min), and detected with corresponding biotinylated secondary antibody (1:500; Jackson Laboratories). Collagen was detected by picrosirius red staining. Necrotic areas were quantified after Masson’s trichrome staining by measuring the surface of acellular and anuclear areas in the atherosclerotic lesions. Apoptosis was detected on 4% PFA-fixed sections using the DeadEnd Colorimetric TUNEL system (Promega). After counterstaining of nuclei with hemalun, images were captured using a Zeiss Axiophot microscope and quantifications were performed by computer image analysis using Image J. The percentage of positive cells was determined by dividing positively stained surface by total lesion surface area. Analysis of PCNA and TUNEL staining was performed by counting positive nuclei over lesion area.

### Magnetic resonance imaging and micro X-ray computed tomography

Whole-body composition was measured using an EchoMRI-700 quantitative nuclear magnetic resonance analyzer. Conscious mice were placed into instrument columns and total body fat, lean mass, body fluids and total body water were measured in a non-invasive manner by ^1^H-magnetic resonance spectroscopy. Values were expressed as percentage of total bodyweight. Mice were sacrificed by CO_2_ inhalation and transferred into the micro-CT Quantum GX (Perkin Elmer). Animals were scanned with a 60 mm field of view at 90 kV and 80 mA over 360 degrees using the whole body scan protocol: 3 scans of 8 seconds automatically stitched together to provide a continuous stack of whole body images. White adipose tissue analysis was performed by segmentation based on the low density (Hounsfield range of values between −50 and −200 HU) of the adipose tissue with Analyze 12.0 (AnalyzeDirect).

### Oral lipid tolerance test and serum lipid analysis

The uptake of long-chain fatty acids was quantified as previously described^40^. In brief, *Panx1* ^*−/−*^ *Apoe* ^*−/−*^ *, Apoe* ^*−/−*^, *Panx1* ^*del*^ *Apoe* ^*−/−*^ and *Panx1* ^*f/fl*^ *Apoe* ^*−/−*^ mice were subjected to an ON fast and subsequently received olive oil (Sigma-Aldrich; 10 μl/g bodyweight) by oral gavage. Blood was obtained by submandibular puncture before (t = 0) and 3 hours after gavage. Total cholesterol, TG and FFA concentrations were measured in mouse sera after HCD or oral lipid tolerance test using a Cobas c111 analyser (Roche Diagnostics).

### Lymphatic drainage, contact hypersensitivity and vessel density

Drainage of interstitial fluids was quantified using Evans Blue injections in the footpad of *Panx1* ^*−/−*^ *Apoe* ^*−/−*^ *, Apoe* ^*−/−*^, *Panx1* ^*del*^ *Apoe* ^*−/−*^ and *Panx1* ^*f/fl*^ *Apoe* ^*−/−*^ mice as described previously^31^. In brief, mice were anesthetized as described above. Then, the mice were injected with 5 μl 5% Evans Blue (dissolved in PBS) in the left footpad using a micro syringe. After 15 min, blood was collected by puncturing the left ventricle and centrifuged 15 min at 5000 rpm (4 °C). Formamide (Sigma) was added to each serum sample (500 μL formamide/200 μL serum) and the mix was incubated ON at 55 °C. Thereafter, presence of Evans Blue in the serum was quantified using a SpectraMax Paradigm Multi-Mode Microplate reader (excitation: 620 nm; emission 680 nm; Molecular Devices). In addition, tail diameters of *Panx1* ^*−/−*^ *Apoe* ^*−/−*^ and *Apoe* ^*−/−*^ were determined using a digital caliper. Mice were briefly anesthetized with 5% isoflurane and the tail diameter was measured at 1 cm from the basis and at 4 cm from the tip. Tail diameters were normalized to mouse weight to correct for size differences between both genotypes.

CH assay was performed as described previously^29^. In brief, a mix of 1:1 acetone/dibutyl phthalate was applied on the skin in the right flank of the mice for 24 hours. Skin-draining LNs were carefully grinded and digested at 37 °C in RPMI containing 1 mg/ml Collagenase IV (Worthington Biochemical Corporation), 40 μg/ml DNase I (Roche) and 2% FBS for 40 min, gently mixing the samples every 20 min. The reaction was stopped by adding a 10% fetal calf serum solution containing 5 mM EDTA (FACS buffer). Samples were then filtered using a 70 μm cell strainer, centrifuged 5 min at 1300 rpm and resuspended in FACS buffer for flow cytometry staining.

To determine lymphatic vessel density, ears were fixed ON at 4 °C and subsequently washed with PBS. Then the inner and outer parts of the dermis of the ear were separated using forceps. After separation, in order to quantify the lymphatic area, whole mount samples were prepared and stained as described before^41^. In brief, samples were blocked ON at 4 °C in blocking buffer (0.5% BSA, 5% donkey serum, 0.3% Triton X-100, 0.1% Sodium Azide in PBS). Subsequently, samples were incubated ON at 4 °C with anti-LYVE-1 antibody (1:100, Abcam) in blocking buffer. Thereafter, samples were washed using washing buffer (0.3% Triton X-100 in PBS) and incubated ON with secondary antibodies (Alexa488, 1:500) in blocking buffer. Next, samples were washed using washing buffer and re-fixed for 48 hours at 4 °C using 4% PFA in PBS. Finally, samples were rinsed using PBS and mounted on a microscopy slide within the wall of three Secure-Seal spacers (Molecular Probes) in order to maintain the three-dimensional structure. Thereafter, LYVE-1 positive areas were visualized using a LSM700 confocal microscope (Zeiss). Three separate recordings were obtained. Z-stacks covering the layer of tissue in which lymphatics are present were obtained during these recordings in order to capture all the lymphatics. After imaging the Z-stacks were converted to a single plain using Fiji using the Z Project plugin. The LYVE-1 positive area was thereafter quantified manually by an observer unaware of the genotype of the mice and was subsequently normalized to the ear area.

### Aortic and lymphatic EC isolation

Thoracic-abdominal aortas from *Panx1* ^*del*^ *Apoe* ^*−/−*^ or *Panx1* ^*f/fl*^ *Apoe* ^*−/−*^ mice were immerged in 100–150 μl PBS on a silicon support, longitudinally opened and pinned. The endothelial side was then delicately scraped with a scalpel blade. The samples were centrifuged 5 min at 1350 rpm and immediately processed for RNA extraction.

LN lymphatic ECs were obtained from skin LN isolated from 8–10 mice and digested in RPMI containing 1 mg/ml Collagenase IV (Worthington Biochemical Corporation), 40 µg/ml DNase I (Roche), and 2% fetal bovine serum gently mixing the samples every 10 min for a total incubation of 30 min. Undigested cells were further digested with 1 mg/ml Collagenase D, and 40 µg/ml DNase I (Roche) for not more than 20 min. The enzymatic reaction was stopped by addition of FACS buffer. Single cell suspensions were negatively selected using CD45 microbeads and magnetic bead column separation (Miltenyi Biotec).

### Antibodies, flow cytometry, and cell sorting

Anti-gp38 (clone 8.1.1), anti-CD31 (clone 390), anti-CD11c (clone N418) and anti-IAb (AF6.120.1) were from BioLegend. Anti-CD16/32 FcyRIII (clone 2.4G2) and anti-CD45 (clone 30F11) were obtained from BD. For purification of lymphatic endothelial cells, enriched CD45-negative cells were FACS-sorted using a MoFlowAstrios (Beckman Coulter). Cells were acquired on a Gallios (Beckman Coulter) and analyzed using FlowJo software (Tree Star).

### BMDM isolation

BMDMs were isolated from *Panx1* ^*del*^ *Apoe* ^*−/−*^ or *Panx1* ^*f/fl*^ *Apoe* ^*−/−*^ mice by flushing femurs and tibiae of posteriors members. Cells were cultured in Iscove’s modified Dulbecco’s medium (IMDM; Gibco) containing Glutamax, 10% fetal bovine serum (Sigma), 100 U/l penicillin, 5 g/l streptomycin (Invitrogen) and 50 μM β-mercaptoethanol. Macrophage colony-stimulating factor (M-CSF) necessary for cell differentiation was provided by supplementing the medium with 30% L929-conditioned medium.

### Real-time PCR

Total RNA from mouse BMDMs, aortic ECs or lymph node ECs was obtained using the NucleoSpin kit (Macherey-Naegel). Reverse transcription was performed using the Quantitect Reverse Transcription kit (Qiagen) and real-time RT-PCR was performed with the ABI Prism StepOnePlus Sequence Detection System (Applied Biosystems) using the TaqMan Fast Universal master mix (Applied Biosystem). Mouse Panx1 or glyceraldehyde-3-phosphate dehydrogenase (GAPDH) primers and probes were purchased from Applied Biosystems. Gene expression was normalized to GAPDH expression.

### Western blot

Proteins were extracted from endothelium denudated aortas, kidneys or livers from C57BL/6 wild-type, *Panx1* ^*−/−*^^38,42^, *Panx1* ^*−/−*^ *Apoe* ^*−/−*^^14,15,43^ *, Apoe* ^*−/−*^^43^, *Panx1* ^*del*^ *Apoe* ^*−/−*^ or *Panx1* ^*f/fl*^ *Apoe* ^*−/−*^ mice and Western blots were performed as described previously^44^. The anti-Panx1 antibody (1:400, Alomone labs) was used ON. Loading controls were performed by probing for GAPDH (1:30000, Millipore).

### Transmigration assay

BMDMs were starved for 14 hours in IMDM without serum and without M-CSF. Cells were washed with PBS and detached by incubating for 5–10 min and flushing with cold PBS containing 0.5 mM EDTA. After washing with IMDM, cells were resuspended in chemotaxis medium (IMDM containing 1% BSA; Sigma-Aldrich) and migration toward 50 ng/ml MCP-1 (CCL2; Peprotec) was determined using Transwells. Briefly, cells were allowed to migrate 90 min (at 37 °C in a humidified 5% CO_2_ atmosphere) through two adjacent compartment separated by a 5 μm polycarbonate filter (Sigma). Cells were added in the upper compartment while the lower compartment was filled with medium (basal migration) or medium containing the chemoattractant MCP-1 (chemotaxis). The percentage of migrated cells was assessed using a Cyan cell analyser (Beckman Coulter).

### Adhesion assay

To assess adhesion of mouse BMDMs, 96-well plates coated with 2.5 μg type 1 collagen (from rat tail, Sigma) per well and blocked with PBS containing 0.5% BSA were used. Assays were performed by plating 5 × 10^4^ CFDA-SE-labeled (Molecular Probes) BMDMs per well. After 30 min incubation, non-adherent cells were removed by washing with PBS and fluorescence emitted by adhering cells was measured (excitation: 492 nm; emission 517 nm) using a SpectraMax Paradigm Multi-Mode Microplate Detection reader (Molecular Devices).

### Statistical analysis

Statistical analysis was performed with Graphpad Prism 6 (v6.0c) and results were expressed as mean ± SEM. Two-group comparisons were performed using Student’s *t-*test. Multiple group comparisons were performed using one-way ANOVA with Bonferroni’s post-test. Differences with a *P* < 0.05 were considered as significant; *P ≤ 0.05; **P ≤ 0.01; ***P ≤ 0.001; ****P ≤ 0.0001.

## Electronic supplementary material

Supplementary information

Movie S1

Movie S2
