# Supplementary material for: Pannexin1 links lymphatic function to lipid metabolism and atherosclerosis
Source: Sci Rep. 2017 Oct 20;7:13706. doi: 10.1038/s41598-017-14130-4 (PMC5651868; doi:10.1038/s41598-017-14130-4)
Supplement: Supplementary file 1 — Supplementary information [file 41598_2017_14130_MOESM1_ESM.pdf]

Pannexin1 links lymphatic function to lipid metabolism and atherosclerosis

Filippo Molica[1], Merlijn J. Meens[1], Juan Dubrot[1], Avigail Ehrlich[1], Christel L. Roth[1], Sandrine Morel[1], Graziano Pelli[1], Laurent Vinet[2,3], Vincent Braunersreuther[1], Osman Ratib[2], Marc Chanson[4], Stephanie Hugues[1], Eliana Scemes[5], Brenda R. Kwak[1,6,*]

Legends to supplementary figures

Figure S1:

(**A**) Cropped Western blots illustrating Panx1 expression in protein extracts (10 µg) from endothelium-denudated thoracic-abdominal aortas (pool of at least 3 aortas per sample) or livers of wild-type, *Panx1*$^{-/-}$, *Panx1*$^{fl/fl}$*Apoe*$^{-/-}$ or *Panx1*^{del}*Apoe*$^{-/-}$ mice. Three bands between 43 kDa and 50 kDa are observed in liver samples, likely corresponding to various glycosylation states of the protein. GAPDH expression was assessed as loading control. (**B-C**) Images illustrating Panx1 expression (in green) in ECs (**B**) of carotid arteries and in epidermis (**C**) of *Panx1*$^{fl/fl}$*Apoe*$^{-/-}$ and *Panx1*^{del}*Apoe*$^{-/-}$ mice. Nuclei were counterstained with DAPI (blue) and elastic laminae with Evans Blue (red). Negative control represents a condition from which the primary antibody was omitted. Scale bars represent 50 µm in **B** and 25 µm in **C**.

Figure S2

Quantification of atherosclerotic lesion extent in thoracic-abdominal aortas of *Panx1*$^{fl/fl}$*Apoe*$^{-/-}$ (white bar) or *Panx1*^{del}*Apoe*$^{-/-}$ (black bar) mice after 5 weeks of HCD (n=9-10).

Figure S3

Sudan-IV staining (**A** and **C**) and quantification of atherosclerotic lesion extent in thoracic-abdominal aortas (**B**) and in aortic sinuses (**D**) of *Apoe*$^{-/-}$ (white bars) or *Panx1*$^{-/-}$*Apoe*$^{-/-}$ (grey bars) mice after 10 weeks of HCD (n=10). Scale bar represents 200 μm.

Figure S4

(**A**) Panx1 mRNA expression was determined by real-time qPCR in bladders of *Apoe*$^{-/-}$ (white bar) and *Panx1*$^{-/-}$*Apoe*$^{-/-}$ (grey bar) mice (n=3). (**B**) Cropped Western blots illustrating Panx1 expression in protein extracts (10 μg) from kidneys of *Apoe*$^{-/-}$ or *Panx1*$^{-/-}$*Apoe*$^{-/-}$ mice. (**C**) Quantification of **B** (n=3). (**D-G**) Representative images of Panx1 immunofluorescent staining (green) performed on carotid atherosclerotic lesions of *Apoe*$^{-/-}$ or *Panx1*$^{-/-}$ *Apoe*$^{-/-}$ mice. Nuclei were stained with DAPI (blue) and elastic laminae were visualized with Evans Blue (red). Scale bar represents 50 μm.

Figure S5

Representative images and quantification of (immuno-)stainings for CD68 (**A-B;** brown signal) and fluorescent staining for α-SMA (**C**-**D;** green signal) performed on low laminar shear stress (LLSS; **A, C left panels**), high laminar

shear stress (HLSS, **A, C middle panels**) and oscillatory shear stress (OSS, **A, C right panels**) regions of casted carotid arteries from *Apoe*$^{-/-}$ (white bars) or *Panx1*$^{-/-}$*Apoe*$^{-/-}$ mice (grey bars) after 10 weeks of HCD (n=5). Scale bar represent 100 μm. Quantification of areas stained by (**E**) the macrophage marker F4/80 in the OSS region as well as of (**F**) the necrotic core and (**G**) the collagen area in the three shear stress regions.

Figure S6

(**A**) Representative images of lymphatic vessels stained for LYVE-1 in the ears of *Apoe*$^{-/-}$ and *Panx1*$^{-/-}$*Apoe*$^{-/-}$ mice. Scale bar represents 100 μm. (**B**) Quantification of LYVE-1^{+} areas in the ears of *Apoe*$^{-/-}$ (white bar) and *Panx1*$^{-/-}$*Apoe*$^{-/-}$ (grey bar) mice (n=6-7).

Movies S1 and S2:

Representative videos of micro X-ray computed tomography scans. Subcutaneous adipose tissue (SAT) and visceral adipose tissue (VAT) can be visualized in green and red, respectively. Movie S1: *Apoe*$^{-/-}$. Movie S2: *Panx1*$^{-/-}$*Apoe*$^{-/-}$.

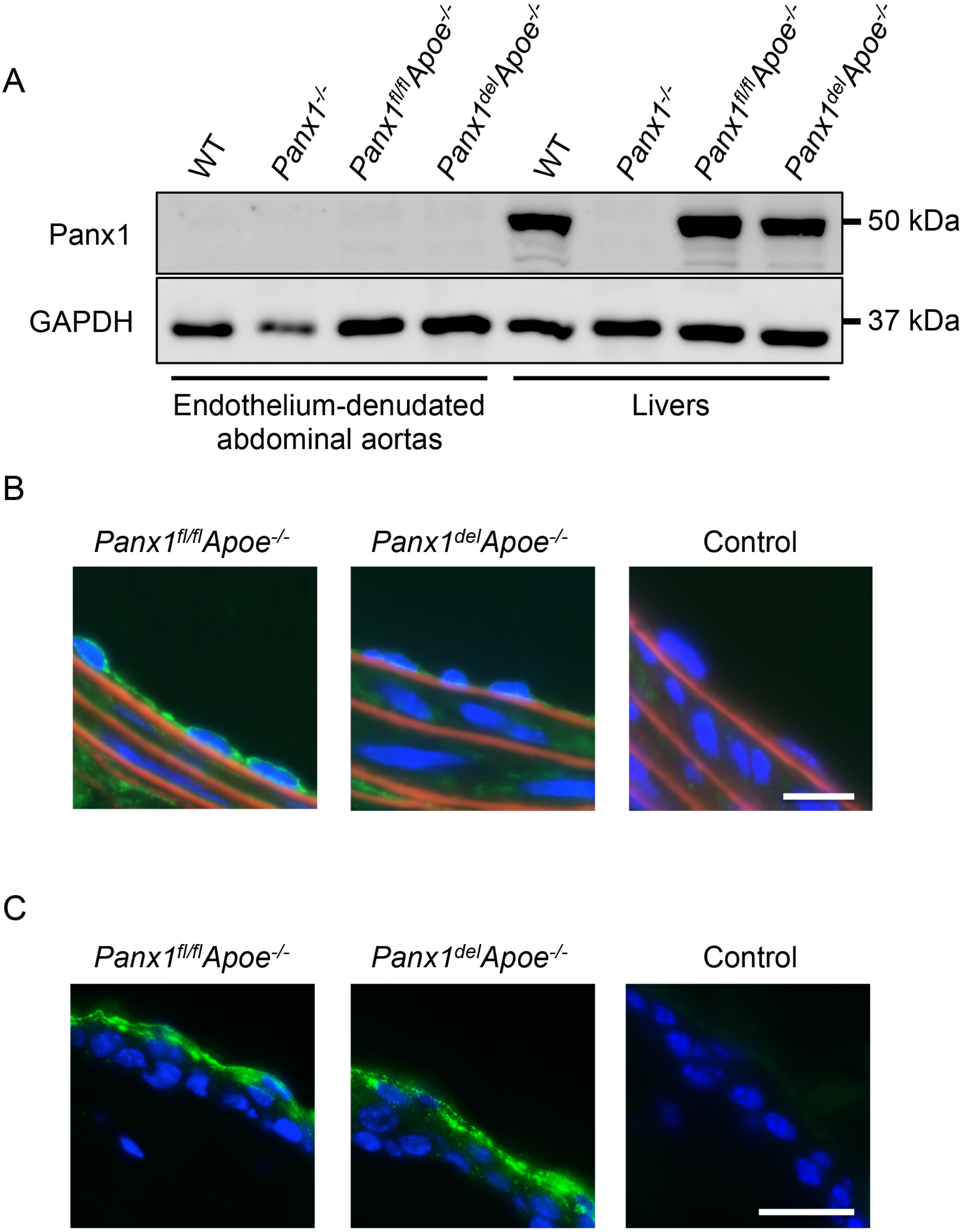
A
WT
Panx1-/-
Panx1fl/fl Apoe-/-
Panx1del Apoe-/-
WT
Panx1-/-
Panx1fl/fl Apoe-/-
Panx1del Apoe-/-
Panx1
GAPDH
50 kDa
37 kDa
Endothelium-denudated abdominal aortas
Livers
B
Panx1fl/fl Apoe-/-
Panx1del Apoe-/-
Control
C
Panx1fl/fl Apoe-/-
Panx1del Apoe-/-
Control

Figure S1

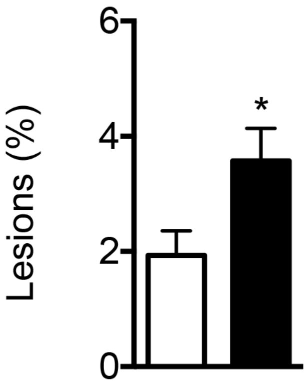

Figure S2

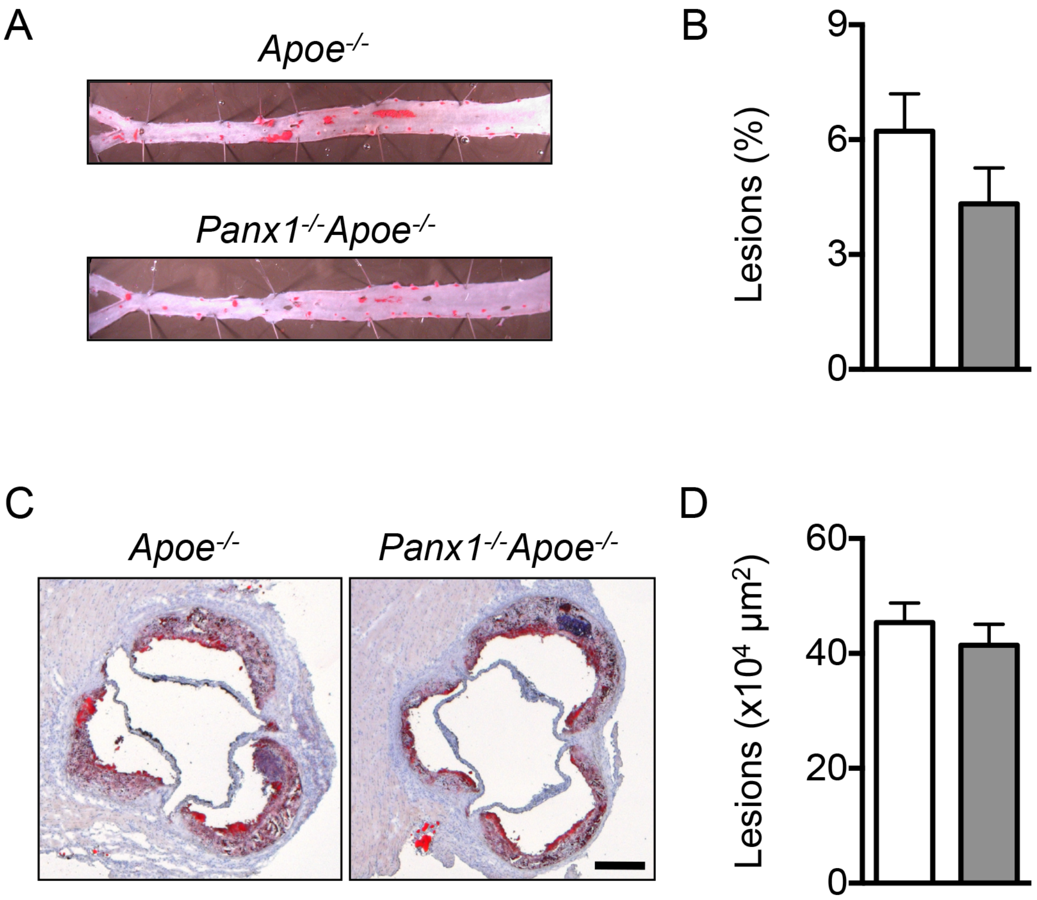
A
Apoe-/-
Panx1-/-Apoe-/-
B
Lesions (%)
9
6
3
0
C
Apoe-/-
Panx1-/-Apoe-/-
D
Lesions (x10^4 µm^2)
60
40
20
0

Figure S3

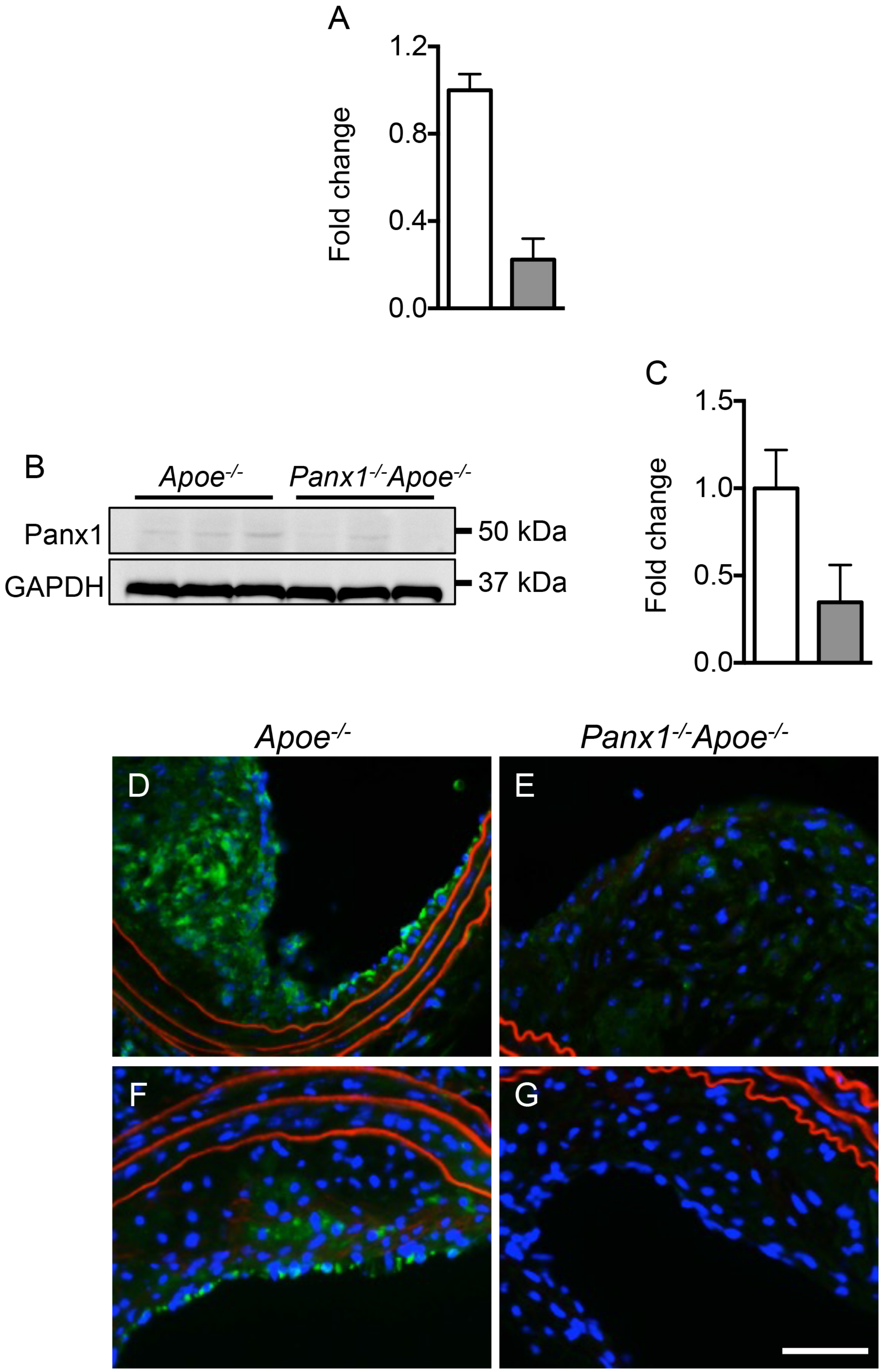
A
Fold change
1.2
0.8
0.4
0.0
B
Apoe-/-
Panx1-/-Apoe-/-
Panx1
GAPDH
50 kDa
37 kDa
C
Fold change
1.5
1.0
0.5
0.0
Apoe-/-
Panx1-/-Apoe-/-
D
E
F
G

Figure S4

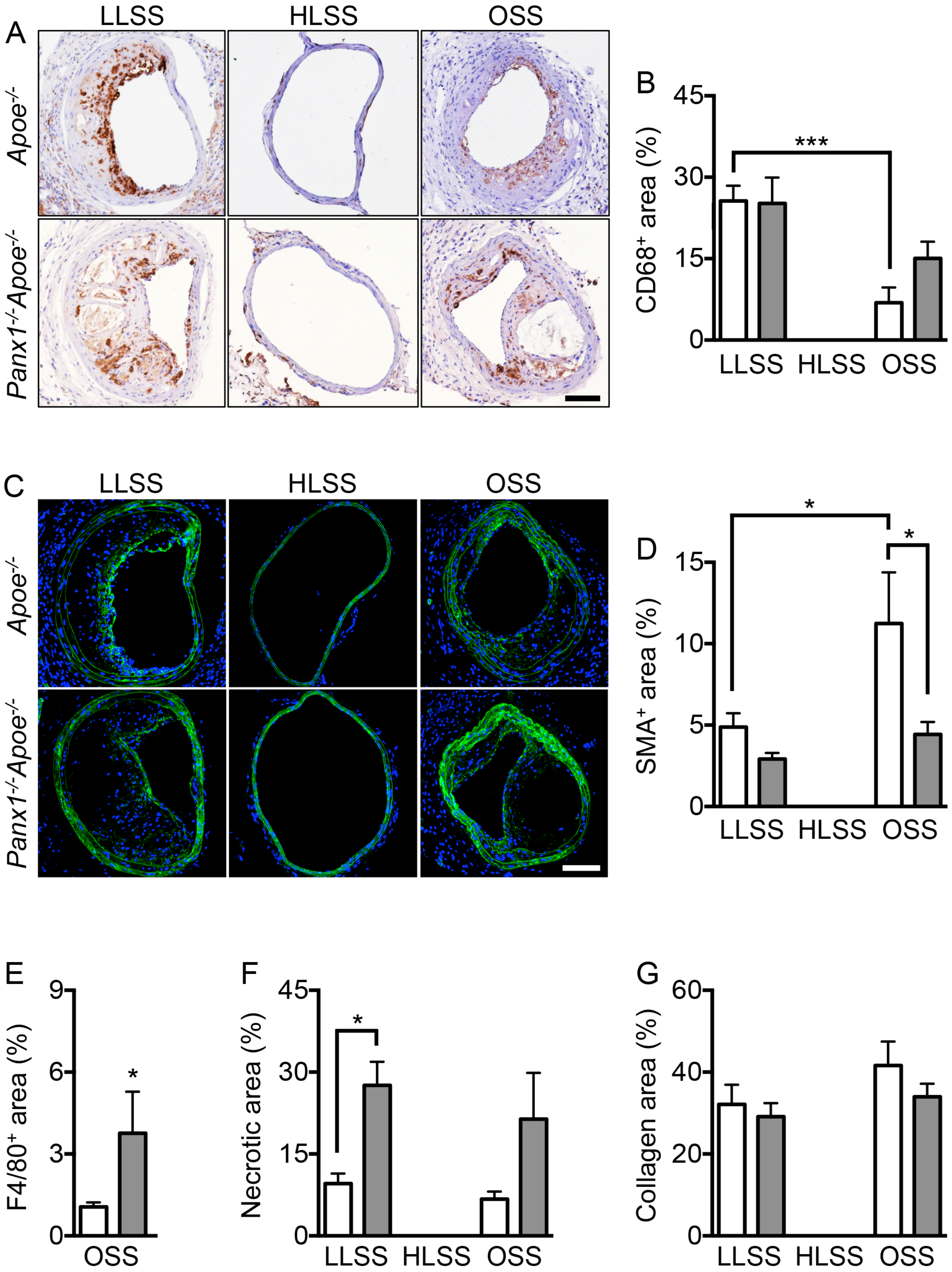
A
LLSS
HLSS
OSS
Apoe-/-
Panx1-/-Apoe-/-
B
45
30
15
0
CD68+ area (%)
***
LLSS HLSS OSS
C
LLSS
HLSS
OSS
Apoe-/-
Panx1-/-Apoe-/-
D
15
10
5
0
SMA+ area (%)
*
*
LLSS HLSS OSS
E
9
6
3
0
F4/80+ area (%)
*
OSS
F
45
30
15
0
Necrotic area (%)
*
LLSS HLSS OSS
G
60
40
20
0
Collagen area (%)
LLSS HLSS OSS

Figure S5

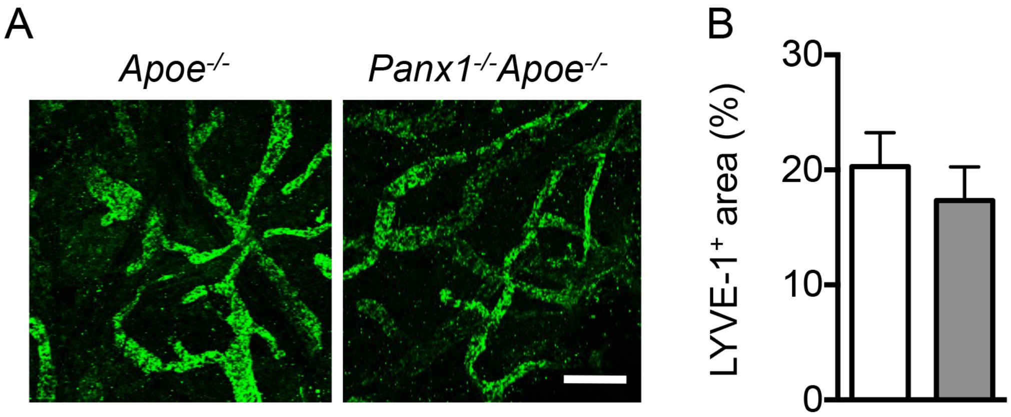

Figure S6
